# Cost-effectiveness of managing HBV reactivation in patients with resolved HBV infection treated with anti-CD20 antibody for B-cell non-Hodgkin lymphoma

**DOI:** 10.1038/s41598-022-10665-3

**Published:** 2022-05-05

**Authors:** Misuzu Fujita, Shigeru Kusumoto, Itsuko Ishii, Tadashi Iwata, Takehiko Fujisawa, Masaya Sugiyama, Akira Hata, Masashi Mizokami

**Affiliations:** 1 Department of Health Research, Chiba Foundation for Health Promotion and Disease Prevention, 32-14, Shinminato, Mihama-ku, Chiba, Chiba 261-0002 Japan; 2 Genome Medical Science Project, National Center for Global Health and Medicine, 1-7-1, Kounodai, Ichikawa, Chiba 272-8516 Japan; 3 Department of Public Health, Chiba University Graduate School of Medicine, Chiba, Chiba Japan; 4 Department of Hematology and Oncology, Nagoya City University Graduate School of Medical Sciences, Nagoya, Aichi Japan; 5 Division of Pharmacy, Chiba University Hospital, Chiba, Chiba Japan; 6 Department of Medical Practice, The University of Tokyo Hospital, Bunkyo-ku, Tokyo, Japan

**Keywords:** Health care economics, Hepatitis

## Abstract

There is no universal recommendation for managing the reactivation of HBV in patients with resolved HBV infection treated with anti-CD20 monoclonal antibodies for B-cell non-Hodgkin lymphoma. This study compared the cost-effectiveness of two commonly used strategies: prophylactic anti-HBV nucleos(t)ide analog therapy (Pro NAT), and HBV DNA monitoring followed by on-demand antiviral therapy (HBV DNA monitoring). Using a decision tree model, the incremental cost-effectiveness ratio (ICER) expressed as cost per quality-adjusted life-year (QALY) gained was calculated. The threshold for cost-effectiveness was set at 5,000,000 JPY, equivalent to 45,662 USD. In a base–case analysis, HBV DNA monitoring was found to be more cost-effective based on the calculation of ICER as 132,048 USD per QALY, a value that far exceeds 45,662 USD. The same results were consistently obtained by a one-way deterministic sensitivity analysis, even after changing each parameter value within the predetermined range. A probabilistic sensitivity analysis with 10,000 simulations also revealed that HBV DNA monitoring is more cost-effective than Pro NAT in 96.8% of cases. Therefore, this study suggests that HBV DNA monitoring is an appropriate managing measure in Japan from a cost-effectiveness perspective.

## Introduction

HBV is detected as covalently closed circular DNA in the hepatocyte nuclei, and remains permanently in an infected individual even after HBsAg becomes negative. In some cases, especially when the host becomes immunologically compromised, HBV replicates itself using the covalently closed circular DNA as a template^1^. Therefore, HBV reactivation occurs not only in HBsAg-seropositive patients but also in patients with resolved HBV infection, who were seronegative for HBsAg but seropositive for the hepatitis B core antibody, during or after cytotoxic chemotherapy or immunosuppressive therapy.

HBsAg-seropositive patients receiving anti-CD20 antibodies (rituximab or obinutuzumab) in combination with chemotherapy for non-Hodgkin lymphoma are a well-known high-risk group for HBV reactivation, with the reported rate of reactivation at 59–80%^1,2^. Current guidelines from various expert organizations consistently recommend prophylactic anti-HBV nucleos(t)ide therapy (Pro NAT) for HBsAg-positive patients before or during immunosuppressive or cytotoxic therapy^3–7^. Meanwhile, the incidence rate for lymphoma patients with resolved HBV infection is relatively low, at approximately 10%^8,9^; however, liver-related death and fulminant hepatitis after HBV reactivation are reported to occur more frequently among such patients than in those with acute hepatitis^10^. Thus, the development of a strategy to manage HBV reactivation in patients with resolved HBV infection is an important issue. Recently, a multi-institutional, prospective observational study in Japan demonstrated that HBV DNA monitoring followed by on-demand antiviral therapy (HBV DNA monitoring) could completely prevent HBV reactivation-related hepatitis in lymphoma patients with resolved HBV infection who received rituximab plus corticosteroid-containing chemotherapy^9^. This result supported a recommendation from the Japan Society of Hepatology (JSH) for patients with resolved infections^7,11^. In contrast, the American Association for the Study of Liver Disease^3^, the American Gastroenterological Association^5^, and the European Association for the Study of the Liver^6^ recommend Pro NAT for patients with resolved HBV infection if they have a high risk of HBV reactivation, such as those receiving anti-CD20 antibody therapy. Further, the American Society of Clinical Oncology recommends either Pro NAT or HBV DNA monitoring for patients with a high risk of HBV reactivation^4^. Although Pro NAT is one of the rational strategies for managing HBV reactivation^12^, it might apply excessive pressure on public health care budgets because it involves administering a nucleic acid analog (NA) to all patients. However, under the HBV DNA monitoring strategy, NA is administered only to patients with evidence of reactivation (the group that experiences reactivation is approximately 10%). Thus, it is important to establish a balance between effectiveness and cost. Notably, insight into how to achieve this balance will be helpful when framing related public policy. To date, three studies have employed a cost-effectiveness analysis to uncover best practices for managing HBV reactivation in lymphoma patients with resolved HBV infection. However, these studies have several drawbacks; for example, they assumed that all patients adhere to the reactivation management strategy. Further, they did not consider the difference of reactivation rate between HBsAb-seropositive and HBsAb-seronegative patients and did not calculate the incremental cost-effectiveness ratio (ICER) per quality-adjusted life-year (QALY)^13–15^.

In response to these gaps, this study compared the cost-effectiveness of the two strategies, Pro NAT and HBV DNA monitoring, for managing HBV reactivation in patients in Japan with resolved HBV infection and receiving anti-CD20 antibody for B-cell non-Hodgkin lymphoma. Further, to evaluate the cost-effectiveness of the two strategies, we developed an easy-to-use Excel sheet wherein the parameter values can be modified and applied according the situation in different countries.

## Results

**Figure 1:**
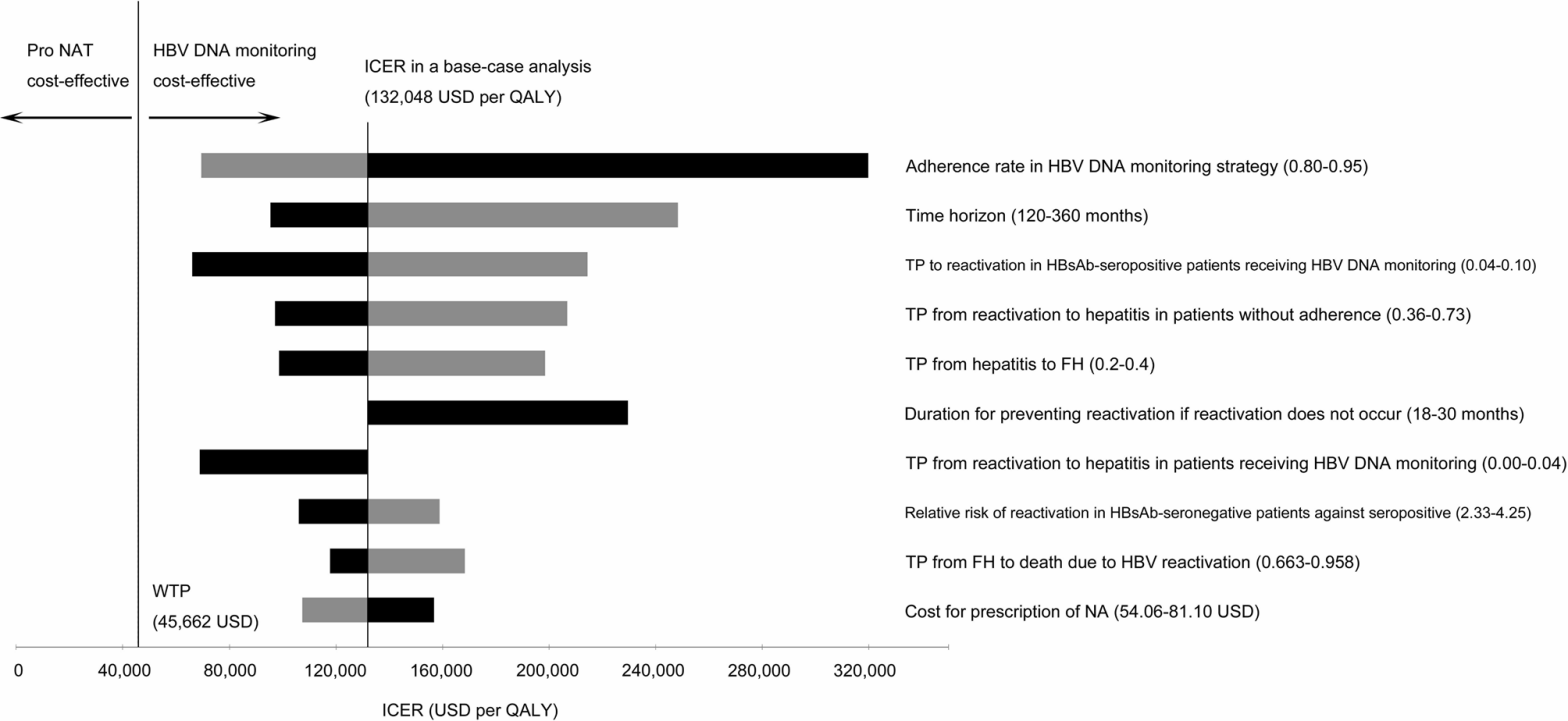
Tornado chart. *FH:* fulminant hepatitis; *HBV DNA monitoring:* HBV DNA monitoring followed by on-demand antiviral therapy; *ICER:* incremental cost-effectiveness ratio; *NA:* nucleic acid analog; *Pro NAT:* prophylactic anti-HBV nucleos(t)ide therapy; *QALY:* quality-adjusted life-year; *TP:* transition probability; *WTP:* willingness to pay. The gray and black bars indicate the ranges when a value was changed to lower and higher, respectively. Costs are expressed as USD. We converted Japanese yen into US dollars using the exchange rate on July 20, 2021: USD 1 = JPY 109.5.

**Figure 2:**
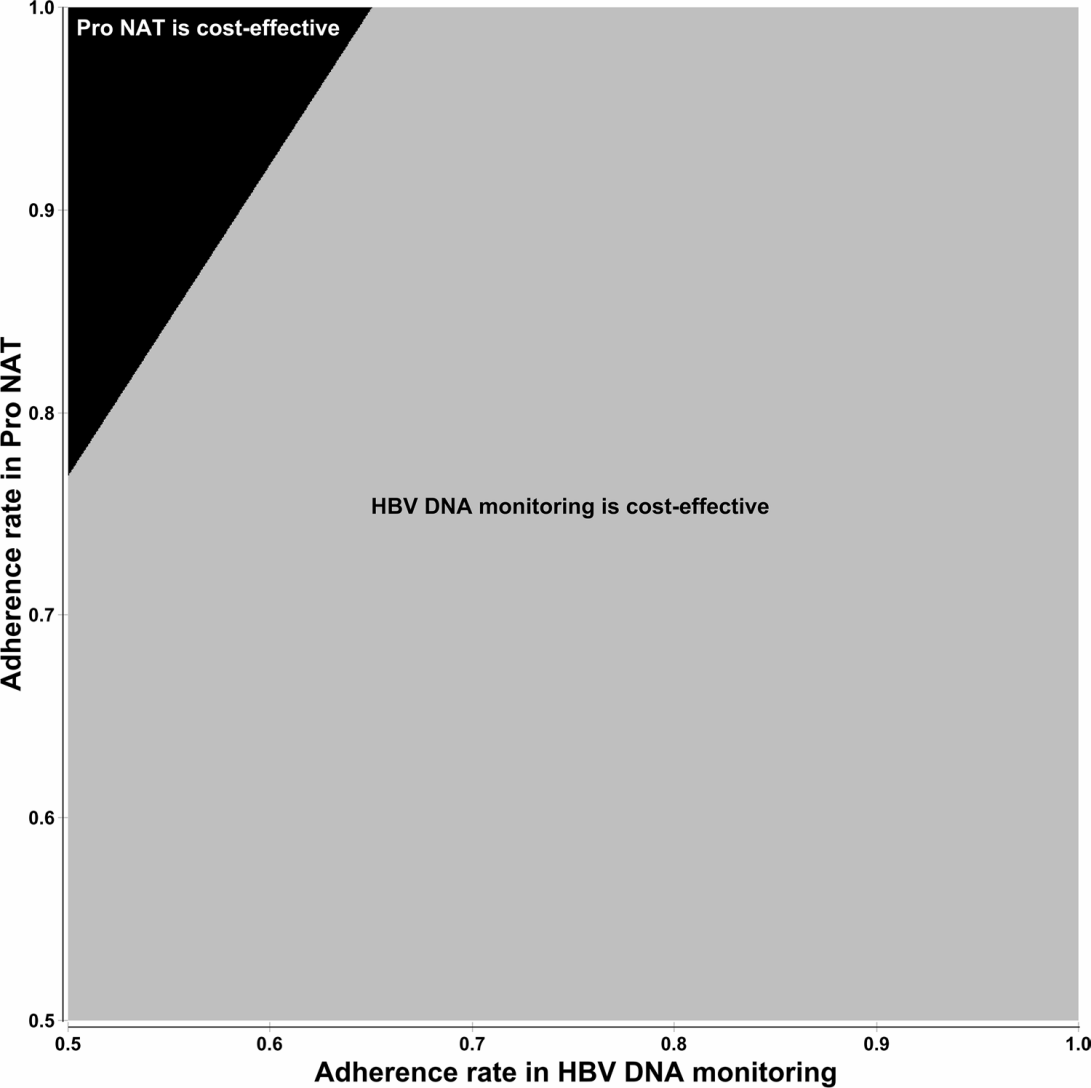
Result of the two-way sensitivity analysis. *HBV DNA monitoring:* HBV DNA monitoring followed by on-demand antiviral therapy; *Pro NAT:* Prophylactic anti-HBV nucleos(t)ide therapy The gray area indicates cases where HBV DNA monitoring strategy was more cost-effective than Pro NAT strategy. The black area indicates cases where Pro NAT strategy was more cost-effective than HBV DNA monitoring strategy.

**Figure 3:**
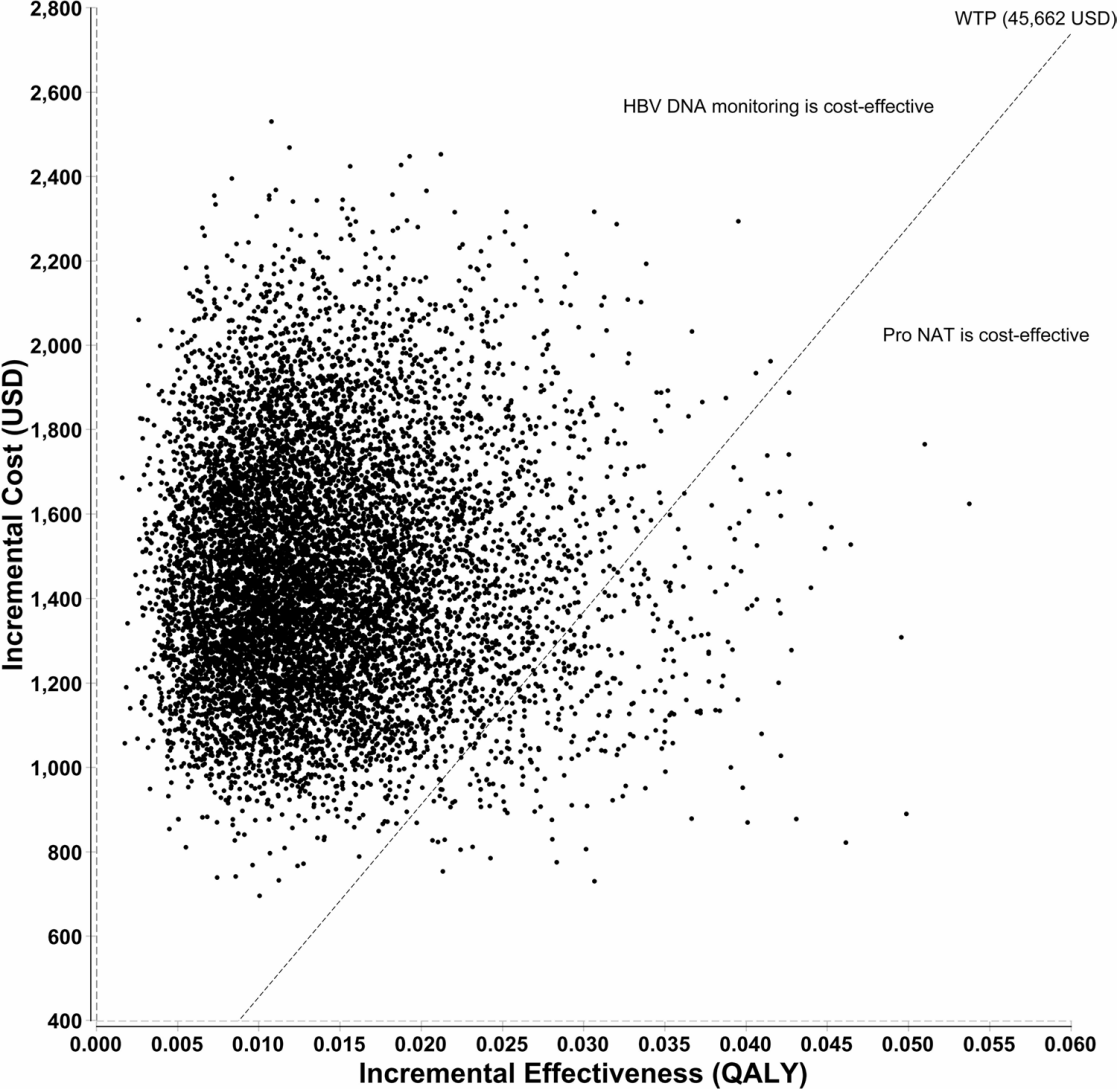
Result of the probabilistic sensitivity analysis. *HBV DNA monitoring:* HBV DNA monitoring followed by on-demand antiviral therapy; *Pro NAT:* Prophylactic anti-HBV nucleos(t)ide therapy; *QALY:* quality-adjusted life-year; *USD:* US dollar; *WTP:* Willingness to pay. Incremental cost was calculated by subtracting cost in HBV DNA monitoring from that in Pro NAT. Incremental effectiveness was calculated by subtracting QALY in HBV DNA monitoring from that in Pro NAT. Dots above and below the dotted lines indicate that HBV DNA monitoring is more or less cost-effective than Pro NAT, respectively. Costs in USD. Japanese yen was converted into US dollars using the July 20, 2021 exchange rate: USD 1 = JPY 109.5.

The results of our base-case analysis are shown in Table 1. The total costs for Pro NAT and HBV DNA monitoring were 4,129 and 2,969 USD, respectively, and the total QALYs were 8.99501 and 8.98622, respectively. ICER was 132,048 USD per QALY, which was much higher than the willingness to pay (WTP) of 45,662 USD. Therefore, the base-case analysis determined that HBV DNA monitoring strategy was more cost-effective than the Pro NAT strategy. A tornado chart is shown in Fig. 1. The parameters with the greatest impact on ICER were (in descending order) “adherence rate in HBV DNA monitoring strategy,” “Time horizon,” and “transition probability (TP) of reactivation in HBsAb-seropositive patients receiving HBV DNA monitoring.” In no occasion was the Pro NAT strategy more cost-effective than HBV DNA monitoring, even when parameter values were changed between predetermined ranges. The variable with the greatest impact on ICER was “adherence rate in HBV DNA monitoring;” hence, a two-way sensitivity analysis was performed as a post-hoc analysis. In the analysis, the adherence rates for Pro NAT and HBV DNA monitoring were changed from 0.5 to 1.0. The result is shown in Fig. 2. In most areas, HBV DNA monitoring was more cost-effective than Pro NAT, depicted by the light gray area in Fig. 2. For Pro NAT to become cost-effective at adherence rates of 1.0, 0.9, and 0.8, the related HBV DNA monitoring rates must be less than 0.65, 0.59, and 0.52, respectively. The result of the probabilistic sensitivity analysis (PSA) with 10,000 simulations is shown in Fig. 3. A dot above the WTP (the dotted line) indicates a case where HBV DNA monitoring is more cost-effective than Pro NAT, whereas a dot below the WTP indicates that Pro NAT is more cost-effective. Of the 10,000 iterations, 9,684 (96.8%) indicated that HBV DNA monitoring was more cost-effective. Based on the results from the scenario analyses (Table 1), ICER was remarkably large when using lamivudine (Zefix tablets) or tenofovir alafenamide (Vemlidy tablets). We developed an easy-to-use Excel file to calculate ICER under the decision tree (Supplementary Excel File S1), in which the values of each parameter, such as cost, the utility values, TP, and duration, can be modifiable according to the situations in each country.

**Table 1:** Results of a base-case analysis and scenario analysis.

Nucleic acid analog and strategy	Cost (USD)	QALY	Incremental cost (USD)	Incremental QALY	ICER (USD per QALY)
**Base case analysis**					
**Entecavir (Entecavir tablets)**					
Pro NAT	4,129	8.99501	1,160	0.00878	132,048
HBV DNA monitoring	2,969	8.98622			
**Scenario analysis**					
**Lamivudine (Zefix tablets)**					
Pro NAT	5,621	8.99501	2,523	0.00878	287,149
HBV DNA monitoring	3,098	8.98622			
**Tenofovir alafenamide (Vemlidy tablets)**					
Pro NAT	7,978	8.99501	4,676	0.00878	532,309
HBV DNA monitoring	3,302	8.98622			

HBV DNA monitoring: HBV DNA monitoring followed by on-demand antiviral therapy; ICER: incremental cost-effectiveness ratio; Pro NAT: prophylactic anti-HBV nucleos(t)ide therapy; QALY: quality-adjusted life-year.

## Discussion

We performed a cost-effectiveness analysis to determine which strategy, Pro NAT or HBV DNA monitoring, was more cost-effective in Japan; we found that HBV DNA monitoring was more cost-effective. This result held even after considering the effect of the uncertainties of several parameters with deterministic and probabilistic sensitivity analyses.

There is no universal recommendation for managing HBV reactivation in patients with resolved HBV infection receiving anti-CD20 antibody therapy for non-Hodgkin lymphoma^3–7,11^. Recently, three studies evaluated the cost-effectiveness of different approaches to managing HBV reactivation in patients with resolved HBV infection undergoing chemotherapy and/or immunosuppressive therapy for lymphoma in Taiwan^15^, China^13^, and Singapore^14^. In these studies, the effectiveness was evaluated as the occurrence of HBV-related deaths^13,15^, liver decompensations^15^, or liver complications^14^, and no event was assumed to occur at all in both the strategies. Under this assumption, these studies performed cost-minimization analyses, in which only costs were compared, and concluded that the HBV DNA monitoring strategy was more cost-effective than Pro NAT^13,14^. We agree with this assumption only if patients adhere strictly to each prevention strategy. Indeed, a meta-analysis in our study indicated that if an HBV DNA monitoring strategy was adopted according to the JSH guidelines, the occurrence of hepatitis after HBV reactivation was 0. However, in reality, perfect adherence in HBV DNA monitoring is unlikely; in fact, the rate was previously reported to be 90.5%^14^. Therefore, in this study, the adherence rates in both strategies were incorporated in the decision tree model. Nevertheless, the main conclusion was consistent with that of the previous studies^13,14^. Additionally, the proportions of HBsAb-seropositive and HBsAb-seronegative patients at the baseline were also set in the model because HBsAb seronegativity is an apparent risk factor for reactivation^8,9,16–18^. These additional settings, derived from meticulous forethought, are strong advantages in our study.

We used QALY in cost-effectiveness analysis for managing HBV reactivation for the first time, which is the preferred effectiveness indicator in economic evaluations for health^19^; as it turned out, ICER, cost per one QALY gained, was obtained. Government authorities in various countries, such as the UK, the Netherlands, Australia, and Sweden, place great importance on ICER determined by QALY when deciding on paying for new pharmaceuticals. Additionally, because QALY is used as an effectiveness indicator, the ICER threshold for evaluating the cost-effectiveness is pre-determined in these countries. For example, the UK has an ICER threshold of 20,000–30,000 GBP per QALY^20^. In the Netherlands, a threshold of 20,000 EUR per QALY is commonly used^21^, and in the Unites States, the threshold is around 50,000–100,000 USD per QALY^22^. Likewise, in Japan, cost-effectiveness analysis has been emphasized recently for policy decision-making. The Central Social Insurance Medical Council in Japan, a body that plays a central role in pharmaceutical price setting, began to apply cost-effectiveness evaluations on medicines and medical devices on a trial basis in 2016 and as a formal system in 2019. This system requires the use of QALY as an effectiveness indicator and the calculation of ICER. Notably, the ICER threshold in Japan is thought to be between 5,000,000–6,000,000 JPY^22,23^; this criterion allowed us to determine which strategies were more cost-effective.

In a cost-effectiveness analysis, several parameters are set in a model, such as costs, TP, and the utility values, and these parameters are usually estimated by a meta-analysis or based on previous studies. Thus, the results of a cost-effectiveness analysis are affected by uncertainty in these parameters and a sensitivity analysis is required. In our one-way sensitivity analysis, the HBV DNA monitoring strategy consistently proved more cost-effective than the Pro NAT strategy even when parameter values were changed. In our two-way sensitivity analysis, adherence rates in both strategies were changed. We searched for points where the result consequently reversed. When the Pro NAT adherence rates were 1.0, 0.9, and 0.8 and the HBV DNA monitoring adherence rates were less than 0.65, 0.59, and 0.52, Pro NAT was more cost-effective. However, according to a multicenter prospective observational study in Japan, in which patients were managed by an HBV DNA monitoring strategy, no hepatitis was found after HBV reactivation^9^. This result suggests that it is not realistic to expect an adherence rate for the HBV DNA monitoring strategy less than 0.65 in Japan. Furthermore, our PSA with 10,000 simulations indicated that HBV DNA monitoring was more cost-effective than Pro NAT in 96.8% of cases. This result suggests that Pro NAT is very rarely cost-effective; indeed, the probability that it will be cost-effective is about 3%, even if parameters are biased in a direction advantageous to Pro NAT. In scenarios where lamivudine (Zefix tablets) or tenofovir alafenamide (Vemlidy tablets) was used instead of entecavir (Entecavir tablets), ICER was remarkably large. From these sensitivity and scenario analyses, the conclusion that HBV DNA monitoring is more cost-effective than Pro NAT in Japan was considered robust.

Kusumoto et al. reported that HBV DNA monitoring appears to be a reasonable strategy for most patients with resolved HBV infections from the perspectives of drug resistance, the risk of delayed reactivation after stopping NA treatment, and the excessive economic burden of prolonged antiviral treatment inherent to the Pro NAT strategy^8^. Additionally, the JSH guidelines recommend HBV DNA monitoring for the patients. Our results support Kusumoto et al.’s assertion and the JSH recommendation from a cost-effectiveness perspective.

Based on the results of one-way sensitivity analysis (Fig. 1), we now discuss the effect of age on cost-effectiveness. The time horizon is set to 240 months (20 years) according to the median age of the target population (about 65 years)^8,9^ and life expectancy in Japan (about 85 years)^24^, as shown in the Method section. Almost all the target patients—that is, lymphoma patients with resolved HBV infection—are assumed to be older patients, although a few young patients are also considered. If young patients escape death by managing HBV reactivation, they will survive longer than the older patients. Therefore, for young patients, the time horizon should be longer. In Fig. 1, black bars indicate the ranges where a value was changed to be higher, that is, the longer the time horizon, the smaller the ICER. As almost all the target patients are older patients, the range of time horizon for sensitivity analysis was set from 120 to 360 months. As a stricter example, we calculated ICER for a time horizon of 720 months, which is equivalent to 25 years of age. The value obtained was 60,845 USD per QALY, which is still higher than the ICER threshold (45,662 USD per QALY); therefore, it can be claimed that HBV DNA monitoring is more cost-effective than Pro NAT in this case.

To obtain the TP to “HBV reactivation” and to “Hepatitis due to HBV reactivation” under the HBV DNA monitoring strategy, we performed a meta-analysis with two subgroups: one with a strict criterion to initiate NA treatment, and the other with a moderate criterion. The results showed that the frequency of hepatitis subsequent to HBV reactivation in the former subgroup was apparently lower than that in the latter subgroup, as suggested by several previous studies^9,15,16^. Under the HBV DNA monitoring strategy, there would be another concern about the risk of developing complications due to a delay in detecting HBV reactivation and, subsequently, initiating the antiviral treatment. However, the results of the meta-analysis revealed that a strict criterion for HBV DNA monitoring could prevent HBV reactivation-related hepatitis completely. This fact supports the JSH guidelines, which recommend HBV DNA monitoring with a strict criterion to detect HBV reactivation^7,11^. The strategy would be feasible in Japan due to the country’s established health insurance system^25^, sufficient number of hospitals^26^, and free access system such that patients are able to visit a medical facility when they want^25^. Indeed, most previous studies with a strict criterion in the meta-analysis were conducted in Japan, and the incidence of hepatitis due to HBV reactivation was estimated to be 0% in this subgroup. However, it is not clear whether such a strategy is feasible in other countries.

There are several limitations to this study. First, some model assumptions included uncertainty. We assumed that death not related to HBV reactivation did not differ between patients with and without reactivation, as previous studies reported no significant difference in the mortality rate between the two groups of patients^17,27^. Conversely, a previous study assumed that mortality depends on HBV reactivation^15^. However, our assumption is reasonable because if HBV reactivation is identified at an early stage by HBV DNA monitoring, preemptive NA treatment can start immediately without withholding lymphoma treatment, allowing the HBV DNA levels to fall below the detection limit in one or 2 months^9,16,28,29^. We also assumed that death not related to HBV reactivation did not depend on age because there is no evidence to support this association^17,27^. The mortality set in the model was derived from a previous study whose subjects were between 60 and 80 years of age^30^. Most of the target population in Japan is thought to be over 60 years old^9^. If the mortality of those under 60 years is different from that of those over 60, the impact would be relatively small, as the number of people under 60 is small. Second, the generalizability of the results is limited. This limitation is unavoidable because the model’s parameters, especially cost and adherence rate, differ across countries. We evaluated cost-effectiveness based on the perspective of the public healthcare payer in Japan; therefore, our results are relevant only for Japan. However, a file to calculate total cost, QALY, and ICER (Supplementary Excel File S1) was developed to overcome this limitation. This file allows the modification of values of each parameter according to the actual situation in each country.

In conclusion, the HBV DNA monitoring approach was found to be more cost-effective than the pro NAT approach for managing HBV reactivation in patients in Japan with resolved HBV infection treated with anti-CD20 monoclonal antibodies for B-cell non-Hodgkin lymphoma. Based on several sensitivity and scenario analyses, this conclusion was thought to be robust.

## Methods

**Figure 4:**
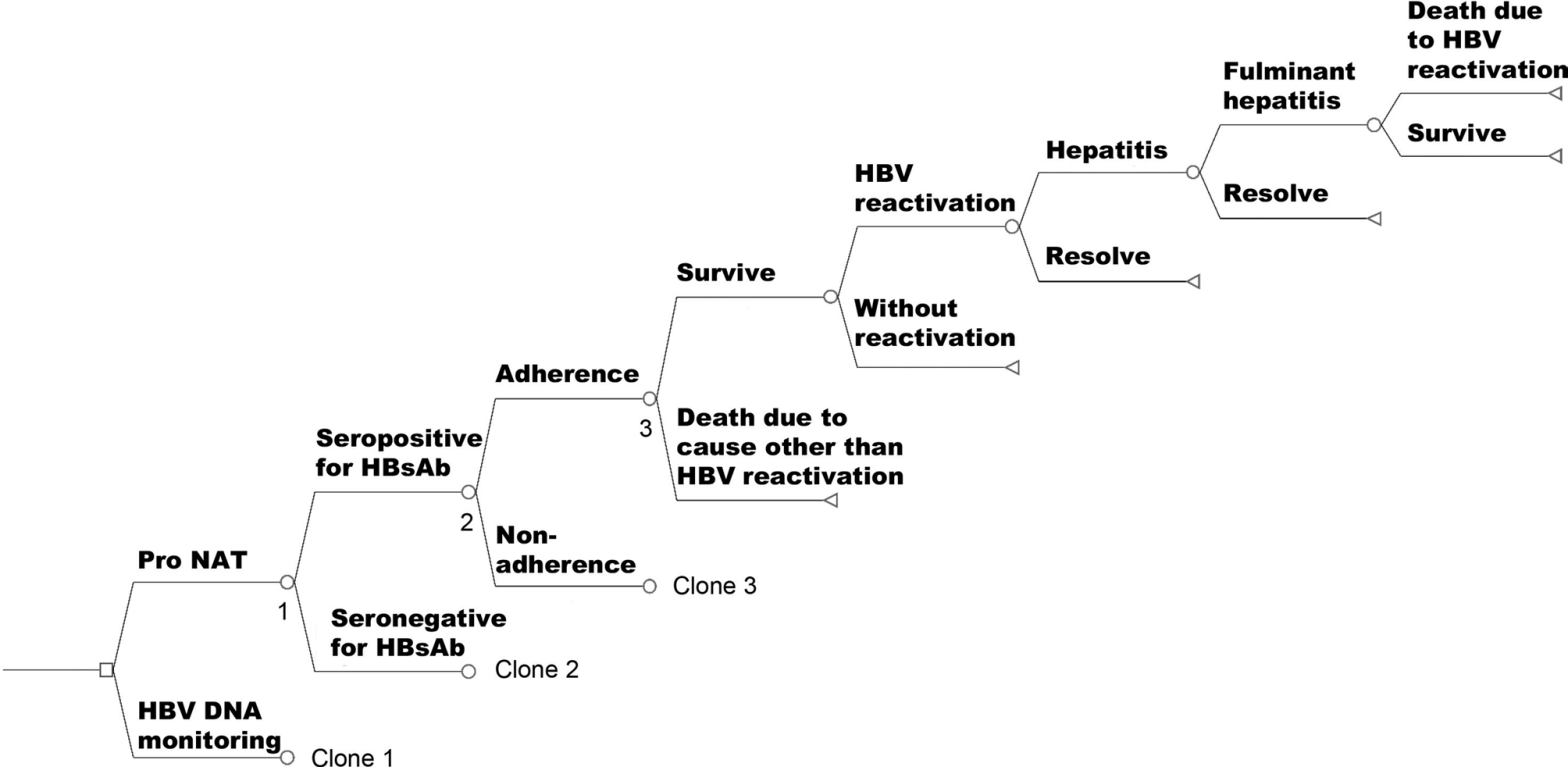
Decision tree. *HBV DNA monitoring:* HBV DNA monitoring followed by on-demand antiviral therapy; *Pro NAT:* Prophylactic anti-HBV nucleos(t)ide therapy.

We conducted a model-based economic evaluation using a decision tree model constructed based on the natural history of HBV reactivation in patients with resolved HBV infection, as shown in Fig. 4. Parameters set in the decision model are shown in Supplementary Table S1. The target population comprised patients in Japan with resolved HBV infection, who were seronegative for HBsAg but seropositive for the hepatitis B core antibody, and were treated with anti-CD20 antibodies for non-Hodgkin lymphoma. The age of patients was assumed 65 years, the median age of the target population^8,9^. The time horizon was determined to be 240 months (20 years), calculated by subtracting the age of the patients from life expectancy of Japan (about 85 years)^24^. The Pro NAT and HBV DNA monitoring strategies were compared. The latter, recommended by the JSH guidelines for patients with resolved HBV infection^7,11^, is widely used in Japan. As we wanted to evaluate the cost-effectiveness of introducing Pro NAT in Japan, HBV DNA monitoring was defined as a comparator and Pro NAT as an alternative strategy. In Pro NAT, patients visit an outpatient clinic every month to undergo the NA treatment, which is initiated simultaneously with the commencement of the treatment for non-Hodgkin lymphoma. Meanwhile, in HBV DNA monitoring, patients visit an outpatient clinic every month to monitor HBV DNA. When HBV reactivation is diagnosed, NA treatment is promptly initiated. The frequency of outpatient visits in both strategies was assumed to be the same, as it is not specified by the JSH^7,11^. The other medical schedules assumed are shown in the Supplementary Method S1. The discount rate was set to 0.02 per year based on the guidance for economic evaluation of health care technologies in Japan^31^. The rate was converted for use in a per-month situation using Eq. (1) $Discount \, rate \, per \, month={(1+Discount \, rate \, per \, year)}^\frac{1}{12}-1$. The occurrence of HBV reactivation in HBsAb-seropositive patients is known to be lower than that in HBsAb-seronegative patients^8,16,32^. Thus, the proportion of patients with HBsAb at the baseline was set in the model accordingly. The proportion was obtained by conducting a meta-analysis of two subgroups, that is, Japan and other countries, using the results of the previous studies, as shown in Supplementary Fig. S1. This was done because the proportion might be different between Japan and other countries. The process of selecting the previous studies is detailed below. As the proportions estimated by the meta-analysis were similar between the two subgroups, an overall estimate was adopted for the model. The adherence rate was set to 0.98 for Pro NAT and 0.90 for HBV DNA monitoring^14^; notably, we took the trend of lower adherence to the latter strategy into account because there are concerns about the feasibility of monitoring in the latter strategy^14^. The health economic analysis plan was not developed in advance.

### TP

Systematic reviews and a meta-analysis were performed to estimate TPs from “resolved HBV infection” to “HBV reactivation”, and from “HBV reactivation” to “Hepatitis due to HBV reactivation” in the HBV DNA monitoring strategy. The target population for the meta-analysis comprised patients with resolved HBV infection who were treated with anti-CD20 antibodies for non-Hodgkin lymphoma and who did not receive a prophylactic NA treatment. To estimate the TPs in HBsAb-seropositive and HBsAb-seronegative patients, articles that reported TPs in both patients were selected. On August 2, 2021, we extracted 53 articles from PubMed and 83 from Web of Science. We evaluated these articles and an additional 42 articles extracted from six meta-analyses. Finally, 11 articles were selected; Supplementary Fig. S2 details the characteristics of these articles.

The picture of progression after HBV reactivation differed based on whether strict or moderate criteria were used for detecting reactivation^16^. Therefore, meta-analyses were performed with both strict and moderate subgroups. The former included articles that used an HBV DNA level threshold of 100 IU/mL or lower to define HBV reactivation, while the latter included articles that used a threshold of more than 100 IU/mL. If the reappearance of HBsAg was an essential condition to detect HBV reactivation, the article was also included in the moderate group.

First, we performed a meta-analysis to estimate TPs to “HBV reactivation” in patients who were HBsAb-seropositive at the baseline (Supplementary Fig. S3). Second, we compared the risk ratios for HBV reactivation in HBsAb-seropositive and HBsAb-seronegative patients (Supplementary Fig. S4). As these estimates did not differ between the two subgroups (p = 0.198 and p = 0.98, respectively), overall estimates were adapted. In detail, TP to “HBV reactivation” in HBsAb-seropositive and HBsAb-seronegative patients were assumed to be 0.06 and 0.06 × 3.15, respectively. The same values were also adopted for patients who did not undergo any treatment to prevent HBV reactivation (non-adherence) because the occurrence of HBV reactivation in those was thought to be the same as patients receiving HBV DNA monitoring. In contrast, the TP from “HBV reactivation” to “Hepatitis” was significantly different between the two subgroups (p < 0.001), as shown in Supplementary Fig. S5: the value in the strict group was 0.00 (95% CI 0.00–0.04), while that in the moderate group was 0.55 (95% CI 0.36–0.73). The meta-analysis was performed without distinguishing between HBsAb-seropositive and HBsAb-seronegative patients because the numbers of HBV reactivation and hepatitis were small. As the JSH guidelines recommends initiating NA treatment if the HBV DNA level is 20 IU/mL or more, the TP in the strict group was adapted to patients with an HBV DNA monitoring strategy; TPs to “Hepatitis” in both seropositive and seronegative patients receiving HBV DNA monitoring were assumed to be 0.00. In contrast, TP to “Hepatitis” in non-adhering patients was adapted to that in the moderate group because the HBV reactivation was thought to be higher. Kusumoto et al.’s study^8^ was applied to the TPs under the Pro NAT strategy (the TP from “HBV resolved infection” to “HBV reactivation,” and the TP from “HBV reactivation” to “Hepatitis due to HBV reactivation”) because it is the only study that reported TPs under the strict criterion. The TP to “HBV reactivation” was assumed to be the same between HBsAb-seropositive and HBsAb-seronegative patients. TP of death not related to HBV reactivation was also set in the model. This probability was determined by a previous study, which reported a 2-year survival rate in patients treated with rituximab plus chemotherapy^30^. This rate was assumed not to depend on age because previous studies reported that overall survival after lymphoma treatment does not significantly vary by age^17,27^. We assumed that if patients do not die within two years, they survive for 20 years (horizon time), which is the difference between the median age of the target population (about 65 years)^8,9^ and the life expectancy in Japan (about 85 years)^24^. The other TPs set in the model are shown in Supplementary Table S1. Meta-analyses were performed using STATA Version 15.0 (STATA LP, College Station, TX).

### Effectiveness

Effectiveness was measured as QALY, to calculate which, the utility values, measured on a scale of 0 (death) to 1 (full health), were set for each situation. The utility value of our target population—lymphoma patients with resolved infection—has not been reported in the previous studies; hence, the value of patients receiving chemotherapy for acute leukemia^33^ was adopted as an alternative because of the similarity of lymphoma and leukemia, which are both considered “blood-related” cancers and are usually treated with chemotherapy. The same value of patients with resolved infection was used for patients with HBV reactivation because the utility value was thought to be unchanged if only reactivation occurred. The utility value in patients with hepatitis or fulminant hepatitis was adopted from that determined by medical specialists using EuroQol 5 dimensions 5-level^34^.

### Costs

The analysis in this study is derived from the perspective of the public healthcare payer, based on the guidance for economic evaluation of health care technologies in Japan^31^. The direct medical costs paid by the government and patients for managing HBV reactivation and treating acute hepatitis and fulminant hepatitis that occurred after HBV reactivation were included. In the base-case analysis, entecavir (in the form of Entecavir tablets, a generic version of Baraclude tablets) was used as an NA because this drug is the cheapest and the most widely used in Japan. The costs set in the model are shown in Supplementary Table S1 and the details of these cost calculations are shown in Supplementary Excel File S2. The cost was calculated in Japanese yen and converted into US dollars using the July 20, 2021 exchange rate: USD 1 = JPY 109.5. The detailed cost assumptions are shown in the Supplementary Method S1.

### Durations

Durations to calculate QALYs and total costs (Supplementary Table S1) were set with reference to previous studies. Fulminant hepatitis is defined as a clinical syndrome characterized by massive liver necrosis within eight weeks of the onset of the initial symptoms^35^. Thus, we assigned one month, which is the middle value, as the duration between the onset of hepatitis and fulminant hepatitis. As no previous study, to our knowledge, has reported the duration between the occurrence of fulminant hepatitis and death, one month was reluctantly assigned. The formulas used to calculate QALY and cost in each terminal node of the decision tree model are shown in Supplementary Tables S3 and S4, respectively.

### A base-case analysis

The ICER was calculated using Eq. (2) $ICER=\frac{{Cost}_{p}-{Cost}_{m}}{{QALY}_{p}-{QALY}_{m}}$. Here, *Cost*_*p*_ and *Cost*_*m*_ are the total costs required for the Pro NAT and HBV DNA monitoring strategies, respectively, and *QALY*_*p*_ and *QALY*_*m*_ are the sums of the utility values for the Pro NAT and HBV DNA monitoring strategies, respectively. A base-case analysis was performed using the values listed in Supplementary Table S1. The cost-effectiveness threshold, determined by WTP, was defined as 5,000,000 JPY^22,23^, which is equivalent to 45,662 USD. If the ICER was less than 45,662 USD per QALY, Pro NAT was judged to be more cost-effective than HBV DNA monitoring; meanwhile, if the ICER was more than the threshold, HBV DNA monitoring was judged to be more cost-effective than Pro NAT. Cost-effectiveness analyses were performed using TreeAge Pro 2019 (TreeAge Software, Inc., MA, USA).

### Sensitivity analysis

The result of the one-way deterministic sensitivity analysis is indicated as a tornado chart, in which the top 10 parameters with the greatest impact on ICER are shown. Ranges for sensitivity analysis are shown in Supplementary Table S1. For PSA, the distributions of the parameters were defined. As the distributions of these parameters were unknown, a triangular distribution was applied. The procedure of the PSA was as follows: 1. The value of each parameter was determined according to the pre-determined distribution; 2. The total cost, QALY, and ICER were calculated using the determined values; and 3. Steps 1 and 2 were repeated 10,000 times and a scatter plot between incremental cost and effectiveness was drawn (n = 10,000).

### Scenario analysis

We calculated ICER using lamivudine (Zefix tablets) or tenofovir alafenamide (Vemlidy tablets) instead of entecavir (Entecavir tablets, a generic version of Baraclude tablets).

### Patient and public involvement statement

Patients, the public, communities, and stakeholders were not involved in the design of the study.

## Supplementary Information

Supplementary Information 1.

Supplementary Information 2.

Supplementary Information 3.

## Data Availability

No dataset was generated during this model-based economic evaluation study.
