# Supplementary material for: Cost-effectiveness of managing HBV reactivation in patients with resolved HBV infection treated with anti-CD20 antibody for B-cell non-Hodgkin lymphoma
Source: Sci Rep. 2022 May 5;12:7365. doi: 10.1038/s41598-022-10665-3 (PMC9072369; doi:10.1038/s41598-022-10665-3)
Supplement: Supplementary file 1 — Supplementary Information 1. [file 41598_2022_10665_MOESM1_ESM.pdf]

Supplementary File

Title: Cost-effectiveness of managing HBV reactivation in patients with resolved HBV infection treated with anti-CD20 antibody for B-cell non-Hodgkin lymphoma

Authors: Misuzu Fujita[1,2,3*], Shigeru Kusumoto[4], Itsuko Ishii[5], Tadashi Iwata[6], Takehiko Fujisawa[1], Masaya Sugiyama[2], Akira Hata[1*], Masashi Mizokami[2*]

Affiliations: [1] Department of Health Research, Chiba Foundation for Heath Promotion and Disease Prevention, Chiba, Chiba, Japan; [2] Genome Medical Sciences Project, National Center for Global Health and Medicine, Ichikawa, Chiba, Japan; [3] Department of Public Health, Chiba University Graduate School of Medicine, Chiba, Chiba, Japan; [4] Department of Hematology and Oncology, Nagoya City University Graduate School of Medical Sciences, Nagoya, Aichi, Japan; [5] Division of Pharmacy, Chiba University Hospital, Chiba, Chiba, Japan; [6] Department of Medical Practice, The University of Tokyo Hospital, Bunkyo-ku, Tokyo, Japan

Table contents

Supplementary Method S1. Cost assumptions....3
Supplementary Table S1. Parameters set in the decision model....5
Supplementary Table S2. Characteristics of the studies selected for meta-analyses....10
Supplementary Table S3. Calculation of QALY in each terminal node....14
Supplementary Table S4. Calculation of cost in each terminal node....15
Supplementary Fig. S1. A forest plot of a meta-analysis for the proportion of HBsAb-seropositive patients in the target population....16
Supplementary Fig. S2. Procedure to select articles for meta-analyses....17
Supplementary Fig. S3. A forest plot of a meta-analysis for the transition probability from “resolved HBV infection” to “HBV reactivation” in the HBV DNA monitoring strategy....18
Supplementary Fig. S4. A forest plot of a meta-analysis for the comparison of the risk ratios of reactivation in HBsAb-seronegative patients and HBsAb-seropositive patients....19
Supplementary Fig. S5. A forest plot of a meta-analysis for the transition probability from “HBV reactivation” to “HBV reactivation-related hepatitis” in the HBV DNA monitoring strategy....20
References....21

Supplementary Method S1. Cost assumptions

- Medical fees under the health insurance system in Japan in April 2020 were used.
- Patients were treated at the University of Tokyo Hospital.
- Patients visit the hospital every month to prevent HBV reactivation in both strategies.
- After the detection of reactivation, patients continue to visit the hospital every month.
- If hepatitis or fulminant hepatitis occurs, patients are hospitalized.
- Given the usual behavior of patients with lymphoma, patients do not receive outpatient treatment on weekends or in the evening.
- Entecavir (in the form of Entecavir tablets, a generic version of Baraclude tablets) is used as a nucleic acid analog (NA), as this drug is the cheapest and the most widely used in Japan.
- Screening tests for HBsAg, a hepatitis B core antibody, HBsAb, HBV-DNA quantification, ALT, and several biochemical tests are performed every visit, as shown in Supplementary Excel File S2.
- NA is provided by pharmacies in the "basic fee for dispensing one" category, in which 90% of pharmacies in Japan are categorized, and patients do not visit pharmacies on weekends or in the evening. However, if a patient is hospitalized, NA is provided by the hospital.
- Since patients have already received treatment for lymphoma before they visit the hospital to manage HBV reactivation, all visits are treated as re-examinations.
- Patients without reactivation receive either Pro NAT or HBV DNA monitoring for 18 months; this was assumed because most HBV reactivations occur within the 12 months after the end of chemotherapy [1-5]. According to the Japanese Society of Hematology guideline for Tumors of Hematopoietic and Lymphoid

Tissues [6], 6–8 cycles of rituximab, cyclophosphamide, doxorubicin, vincristine, and prednisolone (R-CHOP) regimen were recommended for patients with diffuse large B-cell lymphoma, if not otherwise specified. Thus, in this study, we assumed that anti-CD20 antibodies treatment for lymphoma is given in eight cycles to patients (21 days × 8 cycles / 30 days ≈ 6 months). After 18 months, patients received no treatment (i.e., the cost is 0).

- For patients with HBV reactivation, NA treatment is provided over 24 months. After that, no treatment is provided (i.e., the cost is 0).
- Treatments for hepatitis and fulminant hepatitis were performed under hospitalization management.
- If hepatitis and/or fulminant hepatitis occurs and is resolved, NA treatment is provided over 24 months after discharge from the hospital. The treatment is the same as that for HBV reactivation.
- If fulminant hepatitis does not develop after acute hepatitis, then the diagnostic procedure combination (DPC) payment is applied for the first month, and piecework payment is applied thereafter under the health insurance system of Japan.
- If fulminant hepatitis is developed after acute hepatitis, DPC payment is applied for the first and second months, and piecework payment is applied thereafter.
- If fulminant hepatitis is developed, a patient is treated in the intensive care unit for 14 days and undergoes plasma exchange and continuous hemodiafiltration five times [7].

Supplementary Table S1. Parameters set in the decision model

Parameters	Abbreviations[1]	Values	Ranges for sensitivity analysis	References	Explanations
**Time horizon (month)**	TH	240	120–360	1,2,8	This is set according to the median age of the target population and life expectancy in Japan.
**Discount rate (per year)**	Dis	0.02	0.00–0.04	9	The guidance for economic evaluation of health care technologies in Japan.
**Proportions**					
Proportion of patients with seropositive for HBsAb	P1	0.73	0.69–0.77	1-4,10-16	A meta-analysis in Supplementary Fig. S1 Range is set at 95 % CI.
Adherence rate					
Pro NAT	P2	0.98	—		The value is fixed in a one-way sensitivity analysis and PSA. In a two-way sensitivity analysis, the value changed from 0.5 to 1.0.
HBV DNA monitoring	P3	0.90	0.80–0.95	17	The value changed from 0.8 to 0.95 in the one-way sensitivity analysis and PSA. In the two-way sensitivity analysis, the value changed from 0.5 to 1.0.
**Transition probability**					
Resolved infection → Death due to cause other than reactivation	P4	0.3	0.23–0.37	18	Based on the 2-year survival. Range is set at 95 % CI.
Resolved infection → HBV reactivation					
Seropositive for HBsAb					
Pro NAT	P5	0.0213	0.0026–0.0748	1	Range is set at 95 % CI.
HBV DNA monitoring	P6	0.06	0.04–0.10	1-4,10-16	A meta-analysis in Supplementary Fig. S3 Range is set at 95 % CI.

Parameters	Abbreviations[1]	Values	Ranges for sensitivity analysis	References	Explanations
Without adherence (common to both strategies)	P7	0.06	—	[1-4,10-16]	Same as P6.
Seronegative for HBsAb					
Pro NAT	P8	0.0213	—	[1]	Same as P5.
HBV DNA monitoring	P9	×3.15	2.33–4.25	[1-4,10-16]	Relative risk (vs. seropositive for HBsAb)<br>A meta-analysis in Supplementary Fig. S4<br>Range is set at 95 % CI.
Without adherence (common to both strategies)	P10	×3.15	—	[1-4,10-16]	Same as P9.
HBV reactivation → HBV reactivation-related hepatitis					
Pro NAT	P11	0.00	—	[1]	The value is fixed.
HBV DNA monitoring	P12	0.00	0.00–0.04	[1,2,10,11]	A meta-analysis in Supplementary Fig. S5<br>Range is set at 95 % CI.
Without adherence (common to both strategies)	P13	0.55	0.36–0.73	[3,4,12-16]	A meta-analysis in Supplementary Fig. S5<br>Range is set at 95 % CI.
HBV reactivation-related hepatitis → FH	P14	0.3	0.2–0.4	[19,20]	Value is set to approximately the middle value of the references. Range is set at minimum and maximum values of the references.
FH → Death	P15	0.852	0.663–0.958	[21]	Range is set at 95 % CI.
**The utility value**					
Resolved infection	Q1	0.74	0.71–0.77	[22]	The utility value in patients with chemotherapy for acute leukemia.<br>Range is set at 95 % CI.
HBV reactivation	Q2	0.74	—	[22]	Same as Q1.

Parameters	Abbreviations[1]	Values	Ranges for sensitivity analysis	References	Explanations
Hepatitis	Q3	0.529	0.423–0.635	23	The utility value determined by medical specialists using EQ-5D-5L. Range is set at 20% of variation.
FH	Q4	-0.111	-0.133 – 0.000	23	The utility value determined by medical specialists using EQ-5D-5L. Lower range is set at 20% variation and upper range is set at 0.
**Cost (USD)**					Details of cost calculations were shown in Supplementary Excel file S2.
Treatment for preventing reactivation					
Pro NAT	C1	234.06	—		C2+C3
For prescription of NA	C2	67.58	54.06–81.10		Range is set at 20% of variation.
For others	C3	166.48	133.19–199.78		Range is set at 20% of variation.
HBV DNA monitoring	C4	166.48	—		Same as C3
Without adherence (common to both strategies)	C5	0	—		
Treatment for HBV reactivation					
With adherence (common to both strategies)	C6	234.06	—		Same as C1
Without adherence (common to both strategies)	C7	0	—		
Treatment for hepatitis in hospitalization					
Without occurrence of FH after that					
The first month	C8	9,443.56	7,554.85–11,332.27		Range is set at 20% of variation.
The second month or later	C9	5,185.75	4,148.60–6,222.90		Range is set at 20% of variation.
With occurrence of FH after that					
The first month	C10	25,506.76	20,405.41–30,608.11		Range is set at 20% of variation.

Parameters	Abbreviations[1]	Values	Ranges for sensitivity analysis	References	Explanations
Treatment for FH in hospitalization					
The first month	C11	23,929.68	19,143.74–28,715.62		Range is set at 20% of variation.
The second month or later	C12	5,185.75	4,148.60–6,222.90		Range is set at 20% of variation.
Treatment in outpatient after hepatitis and FH are resolved	C13	234.06	—		Same as C1
**Duration of treatment (month)**					
Treatment to prevent reactivation if reactivation does not occur	D1	18	18–30		Detailed is in the text. Range is set with a sufficient width.
Treatment to prevent reactivation if reactivation occurs	D2	4	2.8–11.0	1	Median time to reactivation. Range is interquartile range.
NA treatment after reactivation if hepatitis does not occur	D3	25	12–36	2,11,24	HBV DNA levels fall below the detection limit within one or two months by antiviral therapy. After reactivation is resolved, NA treatment is assumed to provide for 24 months.
Treatment to manage reactivation if hepatitis occurs after reactivation	D4	5	3–7	20	Median time to occur hepatitis. Range is set at minimum and maximum values.
Treatment for hepatitis by hospitalization if it is resolved	D5	2	1–3	25	Acute hepatitis usually calms down in one to three months.
Treatment for hepatitis by hospitalization if FH occurs after hepatitis	D6	1	—	26	Based on the definition of FH.
Treatment for FH by hospitalization if it is resolved	D7	2	1–3	27	A case report: duration of hospitalization for FH is between 40 and 88 days.
Treatment for FH by hospitalization if patient is dead	D8	1	1–2		
NA treatment after discharge from hospital if hepatitis and/or FH occurs	D9	24	12–36		NA treatment is assumed to continue for 24 months after discharge.

Parameters	Abbreviations[1]	Values	Ranges for sensitivity analysis	References	Explanations
**Other duration (month)**					
Survival time if a patient dies due to a cause other than reactivation	D10	24	12–36	[18]	The median follow-up time was 24 months
Duration between reactivation and resolution	D11	1	1–2	[2,11,24]	HBV DNA levels fall below the detection limit within one or two months by antiviral therapy.

EQ-5D-5L: EuroQol 5 dimensions 5-level; FH: fulminant hepatitis failure; HBV DNA monitoring: HBV DNA monitoring followed by on-demand antiviral therapy; IQR: interquartile range; NA: nucleic acid analog; Pro NAT: prophylactic anti-HBV nucleos(t)ide therapy; PSA: probabilistic sensitivity analysis.

Japanese yen was converted into US dollars using the exchange rate on July 20, 2021: USD 1 = JPY 109.5.

Supplementary Table S2. Characteristics of the studies selected for meta-analyses

Study	Country	Period	Design	Disease	Patients (N)	Patients of target population[1] (N)	Patients detected anti-HBs (N)	Definition of HBV reactivation	Definition of hepatitis	Subgroup
Matsue et al. [12] 2010	Japan	Apr 2004 to Apr 2009	Retrospective cohort	B-cell lymphoma	252	56	37	Seroconversion from HBsAg negative to HBsAg positive with or without an increase in HBV-DNA level from baseline (>2.6 log copies/mL)	ALT level > 3-fold higher than the normal upper limit of 2 consecutive determinations 5 days apart in the absence of clinical and laboratory features of acute infection of hepatitis A, hepatitis C, or other systemic infections.	Moderate
Koo et al. [13] 2011	Singapore	2006 to 2009	Prospective cohort	B-cell lymphoma	62	46	33	Reappearance of HBsAg with an increase in HBV DNA levels	N/A Because peak ALT was reported, we defined the ALT level as more than 100 IU/L.	Moderate
Huang et al. [3] 2013	Taiwan	Jun 2009 to May 2012	RCT	CD20+ non-Hodgkin lymphoma	80	39	25	Elevation of HBV viral load to 2,000 IU/mL with two consecutive determinations (>2 weeks apart)	Serum ALT level more than 100 IU/L.	Moderate

Study	Country	Period	Design	Disease	Patients (N)	Patients of target population[1] (N)	Patients detected anti-HBs (N)	Definition of HBV reactivation	Definition of hepatitis	Subgroup
Kim et al. [14] 2013	Korea	Sep 2008 to Feb 2011	Retrospective cohort	B-cell lymphoma	83	83	58	Positive conversion of HBsAg	N/A Frequency of hepatitis was reported despite no definition.	Moderate
Hsu et al [15] 2014	Taiwan	June 2009 to Dec 2011	Prospective cohort	Diffuse large B cell or follicular B cell non-Hodgkin lymphoma	150	150	116	A greater than 10-fold increase of HBV DNA compared with previous nadir levels	Three-fold increase of serum ALT level that exceeded 100 IU/L.	Moderate
Yang et al. [4] 2018	Taiwan	March 2014 to Dec 2015	Prospective cohort	Diffuse large B cell or follicular B cell non-Hodgkin lymphoma	197	197	154	A greater than 10-fold increase of HBV DNA compared with previous nadir levels	Three-fold increase of serum ALT level that exceeded 100 IU/L.	Moderate
Pei et al. [16] 2021	Taiwan	2008 to Dec 2013	Retrospective cohort	B cell lymphoma	128	128	86	HBsAg sero-reversion or HBV viremia >2000IU/ml	A 3-fold or more increase in ALT compared to baseline and > 100 IU/L.	Moderate
Watanabe et al. [10] 2011	Japan	Feb 2007 to Feb 2010	Retrospective cohort	Hematological disease	45	22	13	Re-appearance of serum HBV DNA	An elevation of the serum ALT levels in association with seroconversion of HBV DNA from negative to positive.	Strict

Study	Country	Period	Design	Disease	Patients (N)	Patients of target population[1] (N)	Patients detected anti-HBs (N)	Definition of HBV reactivation	Definition of hepatitis	Subgroup
Seto et al. [11] 2014	Hong Kong	Sep 2011 to Sep 2013	Prospective cohort	Hematologic malignancies receiving rituximab containing chemotherapy	63	63	49	Detectable HBV DNA (≥10IU/mL), regardless of liver biochemistry or HBsAg status	N/A	Strict
Kusumoto et al. [2] 2015	Japan	Sep 2008 to Jul 2011	Prospective cohort	CD20-positive B-cell non-Hodgkin lymphoma	269	268	220	HBV DNA levels of ≥11 IU/mL	Exacerbation of hepatitis clinically with increased HBV DNA levels.	Strict
Kusumoto et al. [1] 2019	Multinational around the world	GOYA trial Jun 2011 to Jun 2014 and GALLIUM trial Jun 2011 to Feb 2014	RCT	B-cell lymphoma	326	225	155	Quantifiable HBV DNA ≥29 IU/mL as follows: 1 result of HBV DNA ≥100 IU/mL, 2 consecutive assessments of ≥29 to <100 IU/mL, or a single assessment between 29 and 100 IU/mL with initiation of NA treatment prior to confirmation	Exacerbation/development of clinical hepatitis with increased HBV DNA.	Strict

N/A: not available; RCT: randomized trial; NA: nucleic acid analog

[1]Target population is patients of non-Hodgkin lymphoma who underwent anti-HBs test at baseline and received anti-CD20 agents, excluding those with prophylaxis nucleotide agent treatment.

The period was that in which patients were enrolled, received chemotherapy regimens, were diagnosed, or began rituximab.

Supplementary Table S3. Calculation of QALY in each terminal node

Node	Common with both strategies
Patients dead due to cause other than reactivation	Q1×D10/12
Patients do not experience reactivation	Q1×TH/12
Patients experience reactivation, but it is resolved	(Q1×D2+Q2*D11+Q1*(TH-D2-D11))/12
Patients experience reactivation and hepatitis, but these are resolved	(Q1*D2+Q2*D4+Q3*D5+Q1*(TH-D2-D4-D5))/12
Patients experience reactivation, hepatitis, and fulminant hepatitis, but these are resolved	(Q1*D2+Q2*D4+Q3*D6+Q4*D7+Q1*(TH-D2-D4-D6-D7))/12
Patients experience reactivation, hepatitis, and fulminant hepatitis, and subsequently die	(Q1*D2+Q2*D4+Q3*D6+Q4*D8)/12

Discount rate was not considered in this table, because the formula is very complex.

Formula was indicated using the abbreviations outlined in Supplementary Table S1.

Supplementary Table S4. Calculation of cost in each terminal node

Node	Pro NAT	HBV DNA monitoring	Without adherence
Patients dead due to cause other than reactivation	C1*if(D1<=D10,D1,D10)	C4* if(D1<=D10,D1,D10)	C5* if(D1<=D10,D1,D10)
Patients does not experience reactivation	C1*D1	C4*D1	C5*D1
Patients experience reactivation, but it is resolved	C1*D2+C6*D3	C4*D2+C6*D3	C5*D2+C7*D3
Patients experience reactivation and hepatitis, but these are resolved	C1*D2+C6*D4+C8*1+C9*(D5-1)+C13*D9	C4*D2+C6*D4+C8*1+C9*(D5-1)+C13*D9	C5*D2+C7*D4+C8*1+C9*(D5-1)+C13*D9
Patients experience reactivation, hepatitis, and fulminant hepatitis, but these are resolved	C1*D2+C6*D4+C10*D6+C11*1+C12*(D7-1)+C13*D9	C4*D2+C6*D4+C10*D6+C11*1+C12*(D7-1)+C13*D9	C5*D2+C7*D4+C10*D6+C11*1+C12*(D7-1)+C13*D9
Patients experience reactivation, hepatitis, and fulminant hepatitis, and subsequently die	C1*D2+C6*D4+C10*D6+C11*1+C12*(D8-1)	C4*D2+C6*D4+C10*D6+C11*1+C12*(D8-1)	C5*D2+C7*D4+C10*D6+C11*1+C12*(D8-1)

The discount rate was not considered in this table, because the formula is very complex.

The formula is indicated using the abbreviations outlined in Supplementary Table S1.

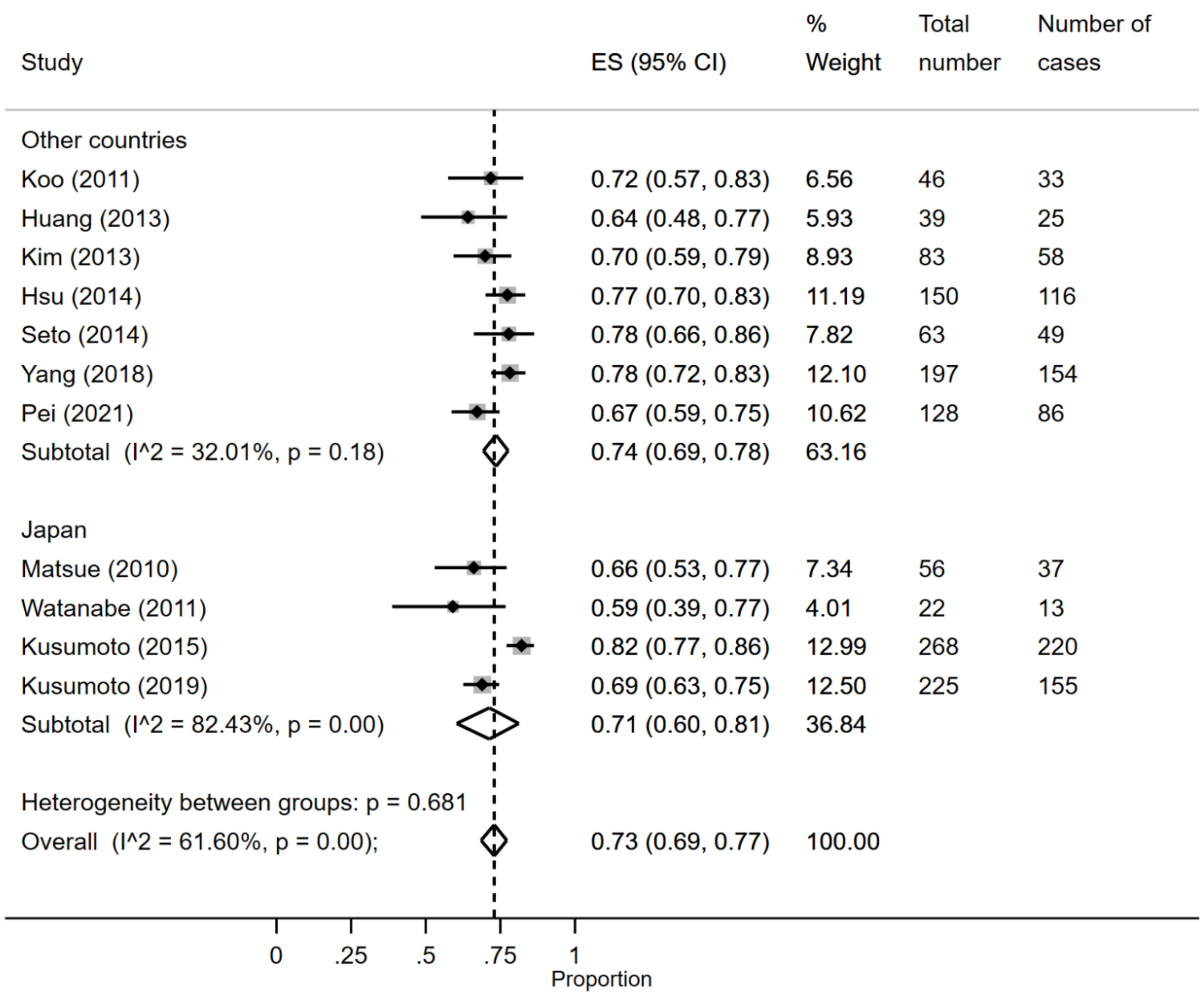

Supplementary Fig. S1. A forest plot of a meta-analysis for the proportion of HBsAb-seropositive patients in the target population

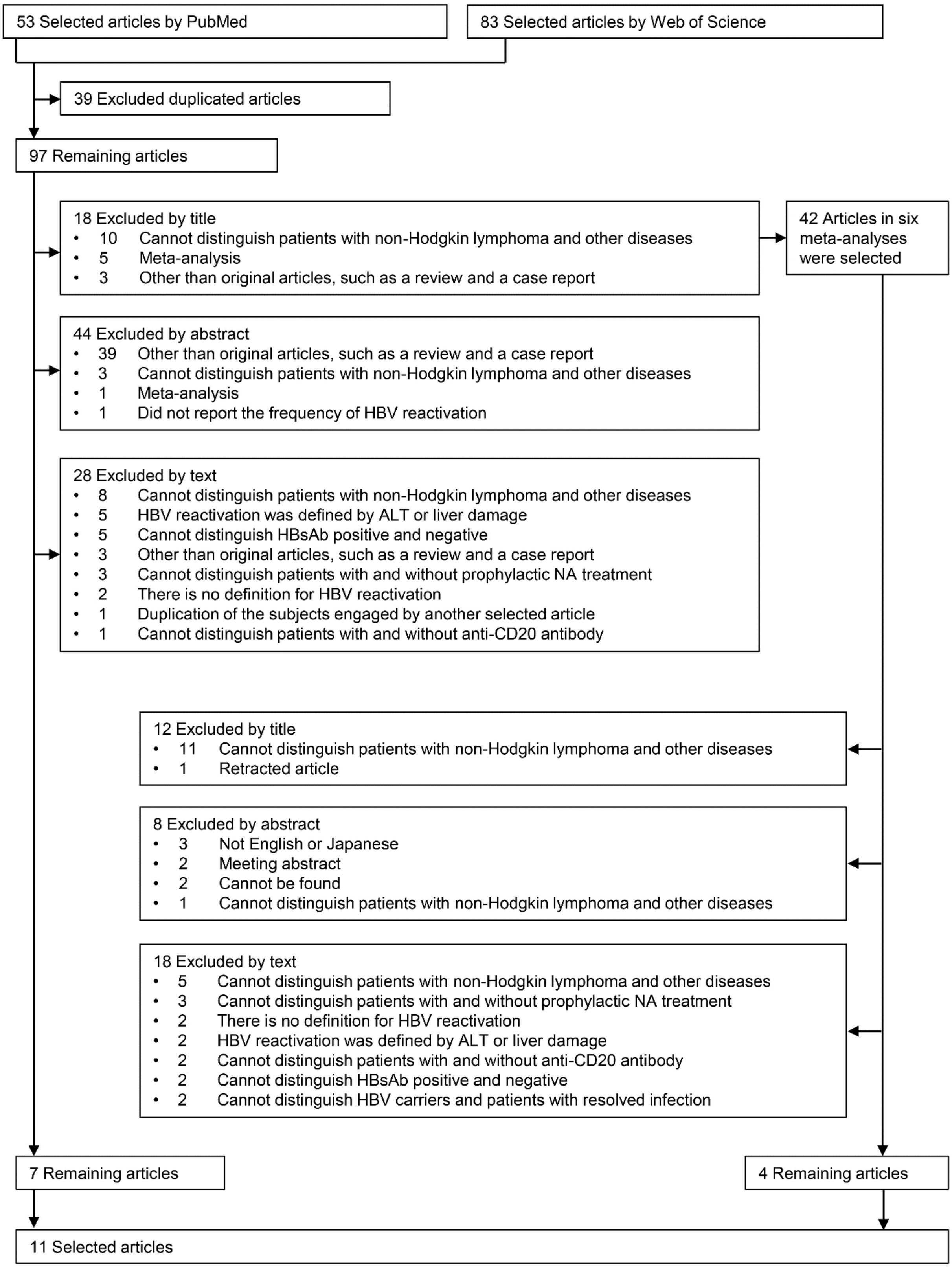

Supplementary Fig. S2. Procedure to select articles for meta-analyses

Extraction condition; (“hbv reactivation”[All Fields] OR “hepatitis b virus reactivation”[All Fields]) AND “lymphoma”[All Fields] AND (“rituximab”[All Fields] OR “r-chop”[All Fields]) AND (“resolved” [All Fields] OR “previous” [All Fields])

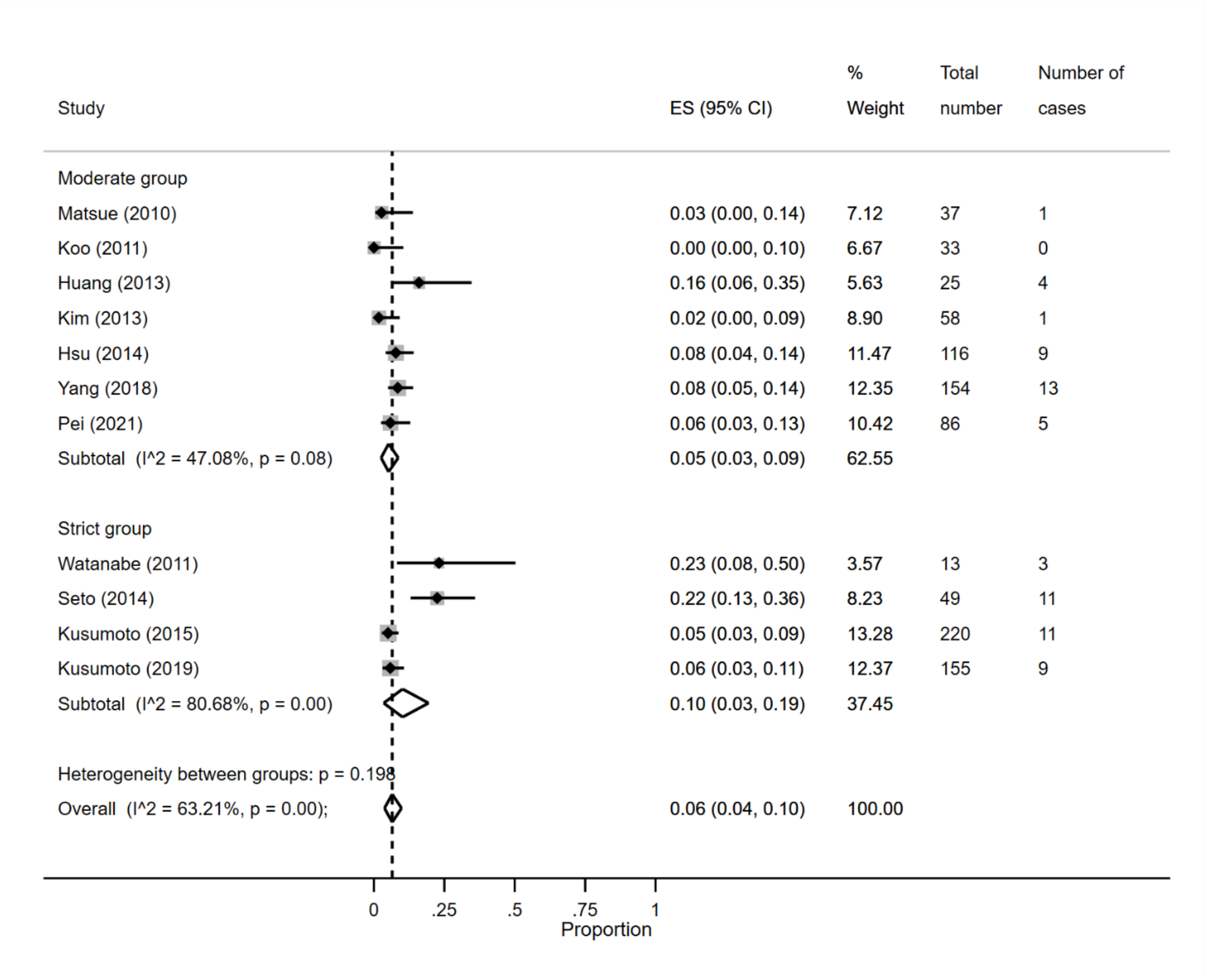

Supplementary Fig. S3. A forest plot of a meta-analysis for the transition probability from “resolved HBV infection” to “HBV reactivation” in the HBV DNA monitoring strategy

Strict group: Threshold of the HBV DNA level to detect reactivation is up to100 IU/ml.

Moderate group: Threshold of the HBV DNA level to detect reactivation is above 100 IU/ml or reappearance of HBsAg was an essential condition to detect HBV reactivation.

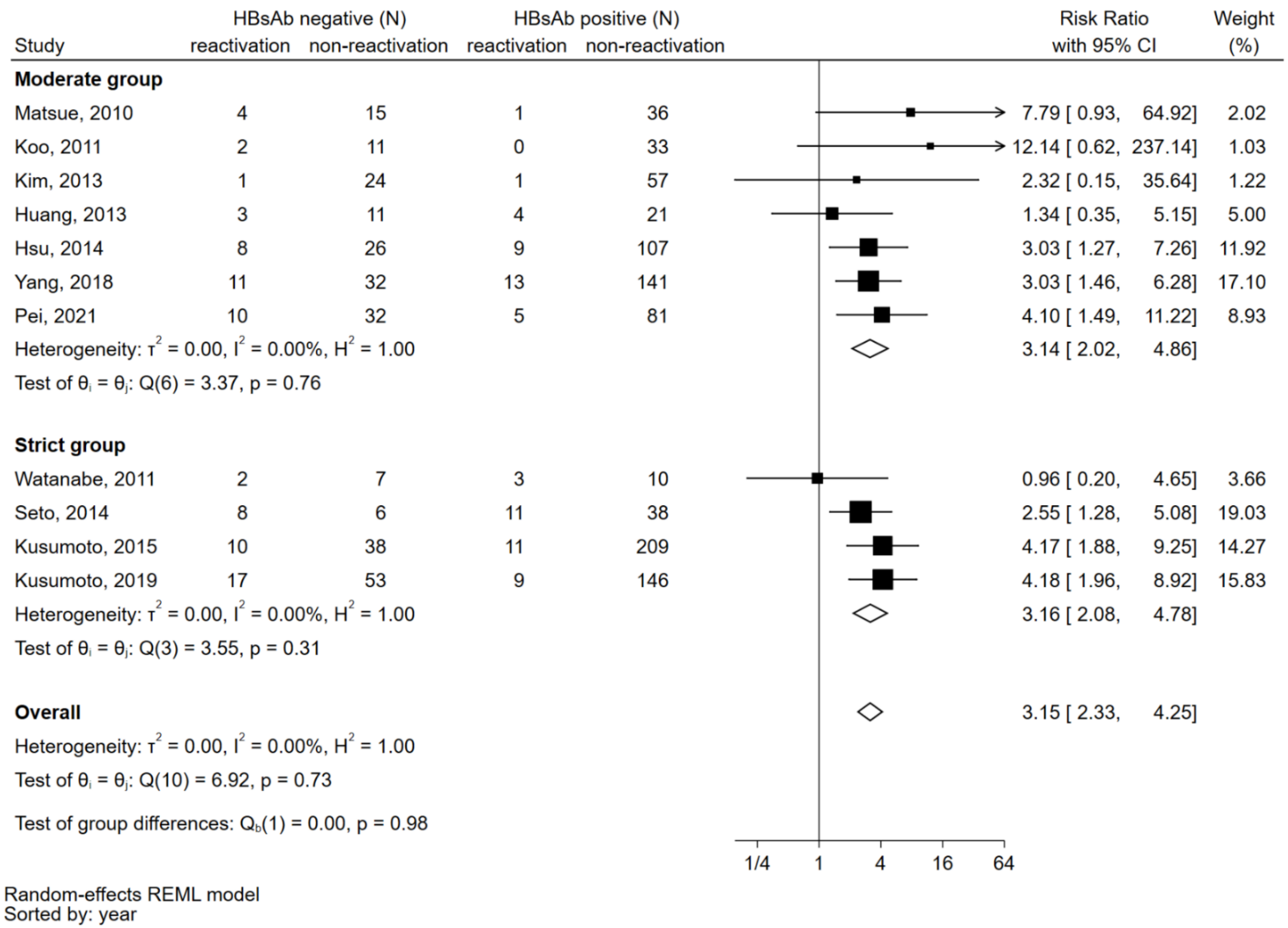

Supplementary Fig. S4. A forest plot of a meta-analysis for the comparison of the risk ratios of reactivation in HBsAb-seronegative patients and HBsAb-seropositive patients

Strict group: Threshold of the HBV DNA level to detect reactivation is up to 100 IU/ml.

Moderate group: Threshold of the HBV DNA level to detect reactivation is above 100 IU/ml or reappearance of HBsAg was an essential condition to detect HBV reactivation.

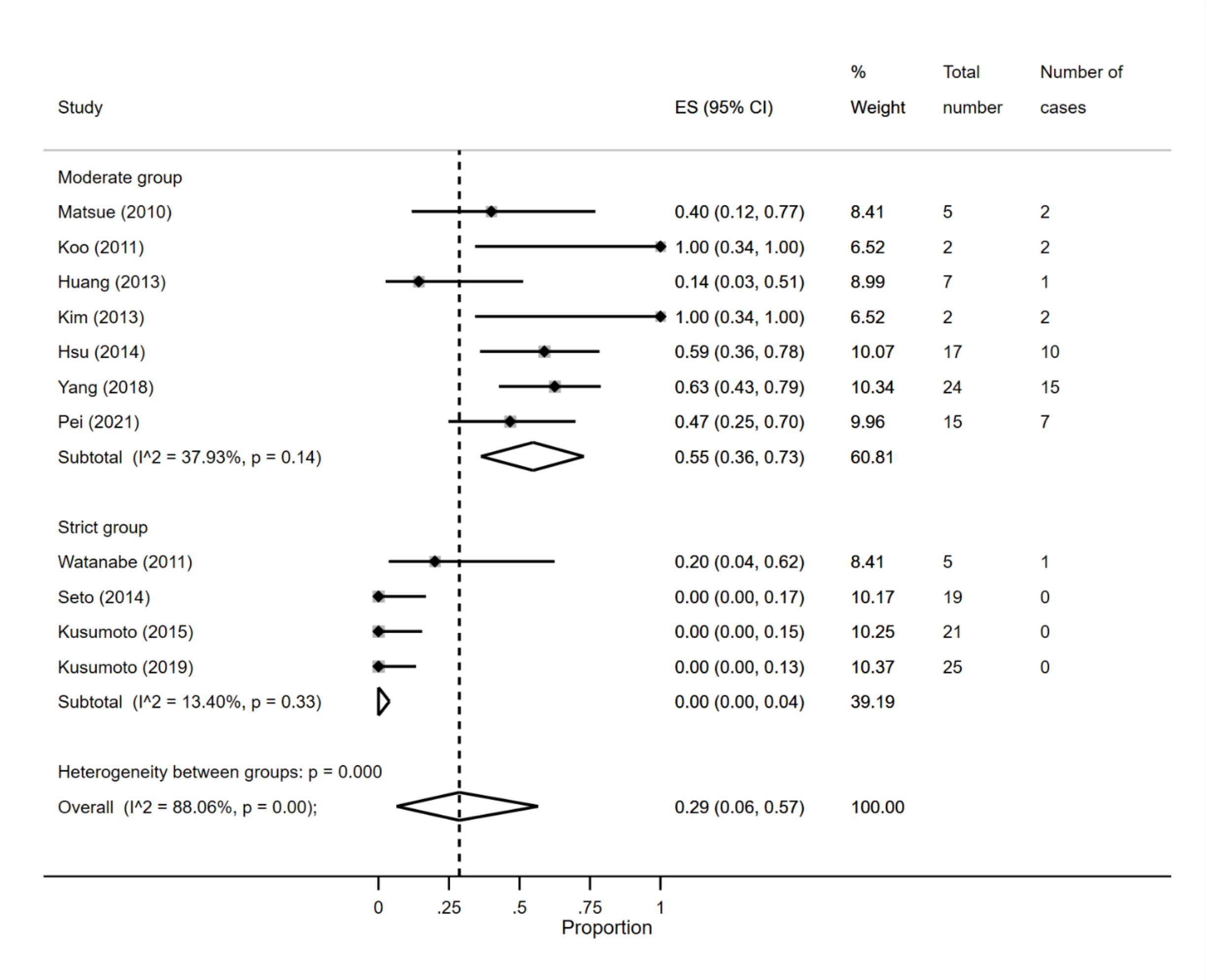

Supplementary Fig. S5. A forest plot of a meta-analysis for the transition probability from "HBV reactivation" to "HBV reactivation-related hepatitis" in the HBV DNA monitoring strategy

Strict group: Threshold of the HBV DNA level to detect reactivation is up to 100 IU/ml.

Moderate group: Threshold of the HBV DNA level to detect reactivation is above 100 IU/ml or reappearance of HBsAg was an essential condition to detect HBV reactivation.

References

1 Kusumoto, S. *et al.* Risk of HBV reactivation in patients with B-cell lymphomas receiving obinutuzumab or rituximab immunochemotherapy. *Blood* **133**, 137-146, doi:10.1182/blood-2018-04-848044 (2019).

2 Kusumoto, S. *et al.* Monitoring of hepatitis B virus (HBV) DNA and risk of HBV reactivation in B-cell lymphoma: a prospective observational study. *Clin. Infect. Dis.* **61**, 719-729, doi:10.1093/cid/civ344 (2015).

3 Huang, Y. H. *et al.* Randomized controlled trial of entecavir prophylaxis for rituximab-associated hepatitis B virus reactivation in patients with lymphoma and resolved hepatitis B. *J. Clin. Oncol.* **31**, 2765-2772, doi:10.1200/jco.2012.48.5938 (2013).

4 Yang, H. C. *et al.* Quantification of HBV core antibodies may help predict HBV reactivation in patients with lymphoma and resolved HBV infection. *J. Hepatol.* **69**, 286-292, doi:10.1016/j.jhep.2018.02.033 (2018).

5 Cho, Y. *et al.* High titers of anti-HBs prevent rituximab-related viral reactivation in resolved hepatitis B patient with non-Hodgkin's lymphoma. *J. Med. Virol.* **88**, 1010-1017, doi:10.1002/jmv.24423 (2016).

6 Nagai, H. Revision of JSH guideline for tumors of hematopoietic and lymphoid tissues 2018: lymphoma. *Rinsho Ketsueki* **59**, 2146-2152, doi:10.11406/rinketsu.59.2146 (2018).

7 Fujiwara, K. *et al.* [Present status of artificial liver support for acute liver failure in Japan]. *Kanzo* **53**, 530-533, doi:10.2957/kanzo.53.530 (2012).

8 Ministry of Health, Labour and Welfare, Government of Japan. *Abridged life tables for Japan 2019* https://www.mhlw.go.jp/english/database/db-hw/lifetb19/dl/lifetb19-06.pdf (2019).

9 Fukuda, T. *et al.* Guideline for economic evaluation of healthcare technologies in Japan. *Journal of the National Institute of Public Health* **62**, 625-640 (2013).

10 Watanabe, M. *et al.* Re-appearance of hepatitis B virus following therapy with rituximab for lymphoma is not rare in Japanese patients with past hepatitis B virus infection. *Liver Int* **31**, 340-347, doi:10.1111/j.1478-3231.2010.02417.x (2011).

11 Seto, W. K. *et al.* Hepatitis B reactivation in patients with previous hepatitis B virus exposure undergoing rituximab-containing chemotherapy for lymphoma: a prospective study. *J. Clin. Oncol.* **32**, 3736-3743, doi:10.1200/jco.2014.56.7081 (2014).

12 Matsue, K. *et al.* Reactivation of hepatitis B virus after rituximab-containing treatment in patients with CD20-positive B-cell lymphoma. *Cancer* **116**, 4769-4776, doi:10.1002/cncr.25253 (2010).

13 Koo, Y. X. *et al.* Risk of hepatitis B virus (HBV) reactivation in hepatitis B surface antigen negative/hepatitis B core antibody positive patients receiving rituximab-containing combination chemotherapy without routine antiviral prophylaxis. *Ann. Hematol.* **90**, 1219-1223, doi:10.1007/s00277-011-1241-0 (2011).

14 Kim, S. J. *et al.* Hepatitis B virus reactivation in B-cell lymphoma patients treated with rituximab: analysis from the Asia Lymphoma Study Group. *Eur. J. Cancer* **49**, 3486-3496, doi:10.1016/j.ejca.2013.07.006 (2013).

15 Hsu, C. *et al.* Chemotherapy-induced hepatitis B reactivation in lymphoma patients with resolved HBV infection: a prospective study. *Hepatology* **59**, 2092-2100, doi:10.1002/hep.26718 (2014).

16 Pei, S. N. *et al.* Role of quantitative hepatitis B surface antibodies in preventing hepatitis B virus-

related hepatitis in patients treated with rituximab. *Leuk. Lymphoma* **29**, 1-8, doi:10.1080/10428194.2021.1948034 (2021).

17 Tan, C. J. *et al.* Clinical and economic evaluation of a surveillance protocol to manage hepatitis B virus (HBV) reactivation among lymphoma patients with resolved HBV infection receiving rituximab. *Pharmacotherapy* **41**, 332-341, doi:10.1002/phar.2508 (2021).

18 Coiffier, B. *et al.* CHOP chemotherapy plus rituximab compared with CHOP alone in elderly patients with diffuse large-B-cell lymphoma. *N. Engl. J. Med.* **346**, 235-242, doi:10.1056/NEJMoa011795 (2002).

19 Umemura, T., Tanaka, E., Kiyosawa, K. & Kumada, H. Mortality secondary to fulminant hepatic failure in patients with prior resolution of hepatitis B virus infection in Japan. *Clin. Infect. Dis.* **47**, e52-56, doi:10.1086/590968 (2008).

20 Hui, C. K. *et al.* Kinetics and risk of de novo hepatitis B infection in HBsAg-negative patients undergoing cytotoxic chemotherapy. *Gastroenterology* **131**, 59-68, doi:10.1053/j.gastro.2006.04.015 (2006).

21 Mochida, S. [Acute Liver Failure in Japan]. *Nihon Naika Gakkai Zasshi* **105**, 1463-1471 (2016).

22 Kurosawa, S. *et al.* Patient-reported quality of life after allogeneic hematopoietic cell transplantation or chemotherapy for acute leukemia. *Bone Marrow Transplant.* **50**, 1241-1249, doi:10.1038/bmt.2015.137 (2015).

23 Hirao, T. *Research on medical economic evaluation of various measures related to viral liver disease* https://mhlw-grants.niph.go.jp/project/23533/1 (2014).

24 Fukushima, N. *et al.* Retrospective and prospective studies of hepatitis B virus reactivation in malignant lymphoma with occult HBV carrier. *Ann. Oncol.* **20**, 2013-2017, doi:10.1093/annonc/mdp230 (2009).

25 Oketani, M. & Tsubouchi, H. [Current status and prevention of fulminant hepatitis due to hepatitis B reactivation]. *Nihon Shokakibyo Gakkai Zasshi* **107**, 1426-1433 (2010).

26 Trey, C. & Davidson, C. S. The management of fulminant hepatic failure. *Prog. Liver Dis.* **3**, 282-298 (1970).

27 Misumi, T. [Plasma Filtration with Dialysis（PDF）for Fulminant Hepatitis Patients before Liver Transplantation]. *Japanese Journal of Apheresis* **36**, 180-183 (2017).
